# Use of navigation to optimize awake prone lateral interbody fusion with posterior decompression and fusion

**DOI:** 10.3171/2026.4.FOCVID25238

**Published:** 2026-07-01

**Authors:** Christian Quinones, Bailey Lupo, Ryan Diaz, Enoch Kim, Stanley Hoang

**Affiliations:** Department of Neurosurgery, LSU Health Shreveport, Louisiana

**Keywords:** awake spine surgery, prone lateral lumbar interbody fusion, spinal anesthesia, stereotactic navigation

## Abstract

This video demonstrates the role of navigation in optimizing awake single-position prone lateral transpsoas interbody fusion with simultaneous posterior decompression and instrumentation under spinal anesthesia. Awake spine surgery is constrained by the duration of spinal anesthesia and demands a coordinated workflow, making navigation essential. The patient was a 77-year-old man with significant cardiac comorbidities and neurogenic claudication from severe L3–4 stenosis. Technical pearls are presented addressing navigational accuracy in the conscious patient and strategies promoting near-simultaneous workflow between the lateral and posterior approaches. Navigation supports both procedural accuracy and workflow efficiency throughout the case.

The video can be found here: https://stream.cadmore.media/r10.3171/2026.4.FOCVID25238

## Transcript

This presentation outlines the technical workflow of an awake single-position prone lateral lumbar interbody fusion with concurrent open posterior decompression and fusion performed under spinal anesthesia. The patient is a 77-year-old male with a history of back pain and neurogenic claudication whose symptoms had progressively worsened despite conservative management. Medical history was significant for coronary artery disease, aortic valve replacement, and pacemaker placement.

### 0:44 Imaging Findings.

Physical examination revealed a right-sided foot drop that happened following hip surgery 2 years earlier. Preoperative T2-weighted lumbar MRI demonstrated severe central canal stenosis at L3–4 attributed to calcified disc herniation and ligament flavum hypertrophy. Scrolling MRI through axial and sagittal images demonstrated additional bilateral foraminal stenosis. A lumbar CT scan revealed disc-based collapse, calcified disc herniation, and bridging osteophytes.

### 1:09 Rationale of the Procedure.

Having failed conservative therapies, a single-position prone lateral transpsoas interbody fusion with posterior decompression and fusion at L3–4 was recommended. The procedure was performed with the patient awake under spinal anesthesia to reduce the risk of intraoperative cardiovascular complications.^1,2^ The goal was to restore disc height and achieve indirect foraminal decompression via lateral interbody cage with direct posterior decompression and fusion.

### 1:33 Risks of the Procedure.

The patient was counseled on the challenges associated with performing the procedure under spinal anesthesia, including a 3-hour operative time limit,^1,3^ potential intraoperative discomfort, and possible conversion to general anesthesia. Additional risks include injury to the lumbar plexus and surrounding vascular structures, as well as instrumentation-related complications.^4^

### 1:52 Benefits of the Procedure.

Spinal anesthesia reduces the risk of intraoperative cardiac events.^1,2^ The single-position approach enables simultaneous anterior and posterior treatment without added operative time. In the prone position, lumbar lordosis enhances, facilitating a safer lateral approach. Triggered EMG remains reliable under spinal anesthesia, which may also reduce postoperative opioid use, shorten hospital stays, and facilitate earlier mobilization.^5–8^

### 2:16 Alternatives.

Alternatives include general anesthesia and other surgical approaches, each of which with its inherent limitations.

### 2:22 Positioning.

Spinal anesthesia was administered with the patient seated, followed by prone repositioning on a Jackson table. The attending surgeon performed the lateral approach from the left, while the assistant resident surgeon performed posterior decompression and instrumentation from the right. Intraoperative navigation was shared between both approaches to maximize efficiency. Necessary equipment included a Jackson table, an O-arm with navigation workstation, lateral retractors, and neuromonitoring. An anesthesiology team experienced in spinal anesthesia was prepared to convert to general anesthesia if needed.

### 2:52 Surgical Steps.

The procedure began with spinal anesthesia administration and confirmation of adequate sensory blockade, initiating the 3-hour operative window. Intraoperative navigation minimized fluoroscopy and enhanced efficiency. A reference frame was secured to the left posterior superior iliac spine, and a bony fiducial was inserted into L3 spinous process to confirm navigation accuracy. Since only one navigated instrument could be active at a time, precise coordination between surgeons was essential. The posterior approach involved pedicle screw placement, decompression, and rod fixation. The lateral approach included EMG-guided transpsoas access, disc preparation, and interbody cage insertion. Each step was coordinated to optimize efficiency and maintain procedural flow.

### 3:34 Images of Spinal Anesthesia.

The operative footage begins with spinal anesthesia administration, with the patient seated. A 25-gauge needle accessed the L3–4 interspace. Intrathecal bupivacaine achieved bilateral motor and sensory blockade, with a sensory level to T5, marking the start of the 3-hour operative window.

### 3:51 Footage of Positioning.

The patient was positioned prone on a Jackson table. Due to a preexisting left frozen shoulder, the left arm board was adjusted based on verbal feedback, an accommodation not possible under general anesthesia.

### 4:02 Navigation Registration.

After sterile draping, a navigation reference frame was affixed to the left posterior superior iliac spine and a 5-mm bony fiducial inserted into the L3 spinous process. An O-arm spin acquired 3D imaging for navigation. Navigation accuracy was confirmed by placing the tip of the navigated probe on the bony fiducial, which appeared as a distinct hyperdense marker atop the spinous process. The dotted green circle on the navigation display confirmed excellent alignment, offering superior reliability over general bony landmarks. This verification was repeated throughout the procedure to ensure navigational accuracy.

### 4:36 Surgical Footage.

The procedure began with posterior exposure via standard subperiosteal dissection. Laterally, dissection continued through the abdominal musculature and transversalis fascia to the retroperitoneal fat. The schematic with the red arrow and circle illustrates the coordinated instrument handoff between surgeons. After entering the retroperitoneal space, the retractor arm was secured. A navigated probe was advanced through the psoas and malleted to breach the lateral osteophyte, achieving disc space access. As the posterior surgeon created a pedicle screw pilot hole, the lateral surgeon sequentially advanced dilators for transpsoas access, which was guided by directionally triggered EMG monitoring to assess proximity to the lumbar plexus. The posterior surgeon then accessed and tapped the pedicle tract under navigation, while the lateral surgeon continued sequential dilation to clear the psoas. As the tap was withdrawn, the lateral retractor was inserted and the pedicle walls palpated simultaneously.

### 5:28 Pedicle Screw Placement.

The right L3 pedicle screw was placed while the lateral surgeon performed the annulotomy. Importantly, both L3 screws were placed prior to disc space distraction to minimize navigation inaccuracy.

### 5:40 Discectomy.

A reference orange line was drawn on the navigation system along the anterior-posterior midline of the disc space to guide interbody trajectory. All disc preparation instruments were advanced along this line. A small navigated Cobb elevator was introduced into the disc space and advanced with malleting, taking care to avoid endplate violation. Once the contralateral annulus was breached, fluoroscopy confirmed endplate integrity. A larger Cobb elevator was then used. Navigation was briefly disrupted when the posterior surgeon inadvertently rotated the instrument’s reference frame into the camera’s field of view, denoted by the yellow circle. This was corrected by repositioning the frame out of the optical path, restoring navigation. The Cobb elevator was malleted across the disc space to disrupt the contralateral annulus, maintaining a controlled trajectory to prevent endplate violation. This is particularly important in a collapsed disc space.

### 6:27 L4 Pedicle Screw Placement.

The left L4 pedicle screw was then placed. Notably, all four pedicle screws were inserted from the right side. This minimized repositioning and preserved workflow efficiency.

### 6:40 Disc Space Preparation.

Four-millimeter and 6-mm distractors were sequentially passed through the disc space to restore disc height. The inset shows the disc space prior to cage insertion.

### 6:51 Intervertebral Cage Placement.

An 8-mm trial was inserted and fluoroscopy confirmed proper positioning and disc height restoration. The posterior surgeon then initiated decompression. A 10-mm interbody cage with 6° of lordosis was selected and malleted into the disc space to ensure proper seating and trajectory.

### 7:10 Patient Discomfort.

During cage insertion, the patient reported abdominal discomfort and nausea. The procedure was paused as the anesthesia team evaluated the patient under the drape, indicated by the yellow arrow. Antiemetics resolved the symptoms and additional local anesthetic was given for comfort.

### 7:28 Procedure Resumed.

The procedure resumed with completion of cage placement as posterior decompression progressed concurrently.

### 7:34 Fluoroscopy.

AP and lateral fluoroscopy confirmed cage position. Final placement was completed 80 minutes from spinal anesthesia initiation. Fluoroscopic images demonstrate optimal cage positioning with no endplate violation. After achieving hemostasis, the lateral retractor was withdrawn. Total retractor time was 28 minutes.

### 7:54 Lateral Closure and Posterior Decompression.

Posterior decompression continued while the lateral incision was closed at 107 minutes. The lateral surgeon then assisted with completing the decompression. Following decompression, rods were placed and set screws tightened.

### 8:09 Fluoroscopy.

Fluoroscopy confirmed appropriate cage and pedicle screw positioning. Liposomal bupivacaine was administered to provide extended postoperative analgesia.

### 8:17 Procedure Completion.

The procedure was completed in 138 minutes.

### 8:21 Summary.

In summary, this patient presented with neurogenic claudication, refractory to conservative treatment, with imaging correlating to clinical symptoms. Given significant cardiovascular comorbidities, the procedure was performed under spinal anesthesia to reduce perioperative risk. An optimized workflow, shared intraoperative navigation, and close anesthesia coordination enabled completion of both approaches within the 3-hour spinal anesthesia window.

### 8:44 Postoperative Imaging and Follow-Up.

Postoperative CT demonstrated restoration of disc and foraminal height, appropriate instrumentation positioning, and no cage subsidence. Postoperative MRI confirmed significant improvement in central stenosis at L3–4. Postoperatively, the patient reported significant improvement in neurogenic claudication with no psoas weakness or lumbar plexus injury. He was discharged on postoperative day 3. At a 2-month follow-up, he demonstrated continued symptomatic improvement with an intact neurological examination and stable imaging.

## Disclosures

The authors report no conflict of interest concerning the materials or methods used in this study or the findings specified in this publication.

## Author Contributions

Primary surgeon: Hoang. Assistant surgeon: Diaz. Editing and drafting the video and abstract: Hoang, Quinones, Lupo, Diaz. Critically revising the work: Hoang, Quinones, Lupo, Kim. Reviewed submitted version of the work: all authors. Approved the final version of the work on behalf of all authors: Hoang. Supervision: Hoang.

## Supplemental Information

### Patient Informed Consent

The necessary patient informed consent was obtained in this study.
